# Development and Validation of a Nomogram to Predict Ventricular Fibrillation During Percutaneous Coronary Intervention in Patients With Acute Myocardial Infarction

**DOI:** 10.31083/RCM37301

**Published:** 2025-07-22

**Authors:** Ruifeng Liu, Xiangyu Gao, Jihong Fan, Huiqiang Zhao

**Affiliations:** ^1^Department of Cardiology, Beijing Friendship Hospital Affiliated to Capital Medical University, 100050 Beijing, China

**Keywords:** acute myocardial infarction (AMI), ventricular fibrillation (VF), percutaneous coronary intervention (PCI), nomogram

## Abstract

**Background:**

Ventricular fibrillation (VF) is a life-threatening complication of acute myocardial infarction (AMI), particularly in patients undergoing percutaneous coronary intervention (PCI). Early identification of high-risk patients is crucial for implementing preventive measures and improving outcomes.

**Methods:**

This retrospective study analyzed clinical, laboratory, and angiographic data from 155 AMI patients to identify predictors of VF during PCI. Variable selection was performed using least absolute shrinkage and selection operator (LASSO) regression, elastic net regression, and random forest. Independent predictors were identified through multivariable logistic regression, and a nomogram was developed and validated to predict VF risk. Model performance was assessed using receiver operating characteristic (ROC) and calibration curves.

**Results:**

Independent predictors of VF included diabetes (OR = 3.676 (1.365–10.668); *p* = 0.012), neutrophil-to-lymphocyte ratio (NLR) (odds ratio (OR) = 1.149 (1.053–1.265); *p* = 0.002), right coronary artery (RCA) intervention (OR = 3.185 (1.088–9.804); *p* = 0.037), Gensini score (OR = 1.020 (1.007–1.033); *p* = 0.003), and absence of beta blockers (OR = 0.168 (0.054–0.472); *p* = 0.001). The nomogram, incorporating these predictors, demonstrated a strong discriminative ability with an area under the ROC curve (AUC) of 0.882 (0.825–0.939) and good calibration (Hosmer–Lemeshow test, *p* = 0.769). The calibration curve showed a strong alignment between predicted probabilities and observed outcomes, with a mean absolute error of 0.033.

**Conclusions:**

This study identified diabetes, NLR, RCA intervention, Gensini score, and absence of beta-blocker use as key predictors of VF during PCI in AMI patients. A nomogram incorporating these factors showed strong predictive performance, aiding clinicians in identifying high-risk patients for targeted preventive strategies.

## 1. Introduction

Ventricular fibrillation (VF) is a life-threatening complication that can occur during acute myocardial infarction (AMI), particularly in patients undergoing primary percutaneous coronary intervention (PCI) [1, 2]. Despite advances in reperfusion therapy and optimal medical management, the incidence of VF during primary PCI for ST-segment elevation myocardial infarction (STEMI) remains substantial, ranging from 4% to 10% [3, 4]. The occurrence of VF is associated with significantly worse clinical outcomes, including increased in-hospital mortality, cardiogenic shock, and long-term adverse cardiovascular events [5, 6]. Early identification of patients at high risk for VF during primary PCI is critical, as it allows for the timely implementation of preventive strategies that may improve clinical outcomes. Several risk factors for VF in the context of AMI have been reported, including diabetes, metabolic derangements, electrolyte imbalances, and the severity of coronary artery disease [7, 8, 9]. However, the relative importance of these factors and their interactions in predicting VF during primary PCI remain poorly understood. The present study aimed to identify clinical, laboratory, and angiographic risk factors associated with the development of VF during primary PCI in patients with AMI. Furthermore, we sought to develop and validate a predictive nomogram to stratify patients at high risk for VF, facilitating early preventive interventions and potentially improving clinical outcomes.

## 2. Research Design

### 2.1 Study Design and Setting

**Fig. 1.**
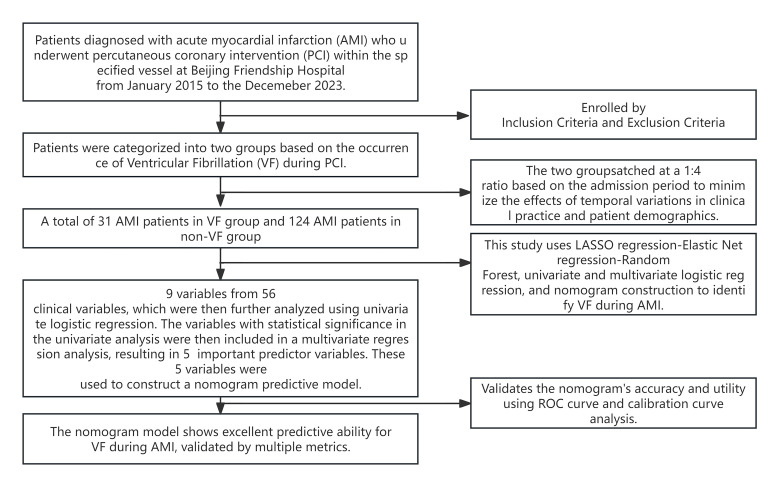
**Flowchart of this retrospective case-control study**. ROS, receiver operating characteristic; LASSO, least absolute shrinkage and selection operator.

This retrospective case-control study was conducted at Beijing Friendship Hospital, utilizing patient data collected between January 2015 and December 2023 (Fig. 1). The study population consisted of 155 patients diagnosed with AMI who underwent PCI on the culprit vessel. The study protocol, as shown in Fig. 1, was approved by the Ethics Committee of Beijing Friendship Hospital (Approval No. 2018-P2-030-01). Informed consent was obtained from all participants before their inclusion in the study. Participants were provided with detailed information regarding the study’s objectives, methodologies, potential benefits, and risks. They were assured of their right to withdraw from the study at any time without penalty. Written consent was obtained to confirm their understanding and agreement to participate.

### 2.2 Grouping Strategy

Patients were divided into two groups based on whether they experienced VF during PCI. The case group consisted of 31 patients who experienced VF during the procedure, while the control group comprised 124 patients who did not experience VF, matched at a 1:4 ratio based on the admission period. This matching was done to minimize potential biases resulting from temporal variations in clinical practice and patient demographics.

### 2.3 Inclusion Criteria

Patients were eligible for inclusion if they met the following criteria: a diagnosis of AMI confirmed by clinical findings, electrocardiogram changes, and elevated cardiac biomarkers; underwent PCI for AMI during the study period; were aged 18 years or older at the time of AMI treatment; and complete medical records, including comprehensive documentation of the PCI procedure and follow-up data.

### 2.4 Exclusion Criteria

Patients were excluded if they had a previous history of VF before the PCI procedure, required immediate cardiac surgery (e.g., coronary artery bypass grafting), lacked consent for the use of medical data for research purposes, or had incomplete data, including missing critical baseline demographic, clinical, or procedural details. Other exclusion criteria included diagnoses of cardiomyopathy, valvular heart disease, chronic heart failure, aneurysms in other vessels, collagen tissue diseases, vasculitis, syphilis, chronic obstructive pulmonary disease, pulmonary hypertension, early menopause, hepatic disease, renal failure, malignancy, local or systemic infection, history of infection (<3 months), or other acute or chronic inflammatory diseases.

## 3. Collected Data

### 3.1 Clinical Characteristics

Baseline data were extracted from medical records and included demographic information (age, sex), medical history (e.g., coronary heart disease, diabetes, and other conditions), smoking and alcohol consumption history, family history of hypertension, diabetes, and coronary heart disease, as well as medications taken before admission or after discharge. The body mass index (BMI) was calculated as weight in kilograms divided by height in meters squared (kg/m^2^).

### 3.2 Biochemical Indicators

Blood samples were collected from the elbow vein on the morning after admission and analyzed in the hospital laboratory. Serum levels of the following were measured: alanine aminotransferase (ALT), aspartate aminotransferase (AST), serum creatinine, urea nitrogen, total cholesterol (TC), triglycerides (TG), high-density lipoprotein cholesterol (HDL-C), low-density lipoprotein cholesterol (LDL-C), and other components were measured. For AMI patients, serum concentrations of troponin I (TnI), myoglobin (Myo), creatine kinase-MB (CK-MB), and N-terminal pro-B-type natriuretic peptide (NT-proBNP) were measured at admission, and 12-hour intervals during the first five days after symptom onset.

### 3.3 Echocardiography and Coronary Angiogram Analysis

Transthoracic echocardiography was performed after hospital admission and at a median of five days post-AMI. All images were analyzed by a single investigator who was blinded to the clinical data. Coronary angiography was performed via radial or femoral artery access, and images were reviewed by two independent cardiologists.

### 3.4 PCI Procedure

Most patients with STEMI underwent emergency PCI as part of reperfusion therapy within 12 hours of symptom onset. For non-STEMI patients, initial antithrombotic therapy was administered, followed by coronary angiography (delayed PCI) within the first week.

### 3.5 Gensini Scoring

The Gensini scoring system was used to evaluate the severity of coronary stenosis. Stenotic diameters were scored as follows: <25% = 1 point, 25–49% = 2 points, 50–74% = 4 points, 75–89% = 8 points, 90–99% = 16 points, and total occlusion = 32 points. Scores were multiplied by coefficients based on lesion location, and the total score represented the degree of coronary artery stenosis.

### 3.6 Analytical Approach

All statistical analyses were conducted using R software (version 4.2.2, released October 31, 2022; R Foundation for Statistical Computing, Vienna, Austria; https://www.r-project.org/). Continuous variables were summarized as means and standard deviations, while categorical variables were described as frequencies and percentages. Independent sample *t*-tests or Mann-Whitney U tests were used to compare continuous variables, depending on data distribution. Chi-square or Fisher’s exact tests were applied for categorical variables.

### 3.7 Variable Selection

To identify predictors of VF, three variable selection methods were employed. Least absolute shrinkage and selection operator (LASSO) regression applied an L1 penalty to regression coefficients, shrinking some to zero to identify the most relevant predictors, thereby reducing overfitting and addressing multicollinearity. Elastic net regression combined L1 (LASSO) and L2 (Ridge) regularization to handle multicollinearity and retain correlated variables, with the optimal lambda determined via cross-validation. Random forest evaluated variable importance using metrics like mean decrease accuracy (MDA), selecting the top 30 variables based on their importance. The final variables were determined by taking the intersection of the three methods and were subsequently used for logistic regression analysis.

### 3.8 Logistic Regression and Nomogram Development

Univariate and multivariate logistic regression analyses were performed to identify independent predictors of VF during PCI. Variables with a *p*-value < 0.1 in univariate analysis were included in the multivariate model. Odds ratios (ORs) and 95% confidence intervals (CIs) were calculated to quantify associations. A predictive nomogram was constructed based on multivariate analysis results to estimate the probability of VF during PCI. The nomogram was validated using the receiver operating characteristic (ROC) curve to assess discriminative ability and the calibration curve to evaluate agreement between predicted probabilities and observed outcomes. All statistical tests were two-sided, and *p*-values < 0.05 were considered statistically significant.

## 4. Result

### 4.1 Baseline Characteristics

Table 1A highlights significant differences in baseline characteristics between patients with ventricular fibrillation (VF group, n = 31) and those without VF (non-VF group, n = 124). Notably, the VF group had a higher prevalence of diabetes (61.29% vs. 37.10%, *p* = 0.015) and a significantly lower prevalence of dyslipidemia (35.48% vs. 65.32%, *p* = 0.002). Biochemical markers such as ALT (46.00 (21.00, 64.00) vs. 22.00 (14.00, 35.25), *p* = 0.002) and AST (126.00 (29.20, 352.30) vs. 35.05 (19.18, 93.85), *p* = 0.001) were markedly elevated in the VF group, suggesting potential liver involvement. Additionally, urea nitrogen levels were higher in the VF group (5.76 (5.05, 7.74) vs. 5.19 (3.91, 6.50), *p* = 0.008). The VF group also exhibited significantly higher rates of metabolic acidosis (25.81% vs. 2.42%, *p* < 0.001) and hypokalemia (45.16% vs. 11.29%, *p* < 0.001), both of which are critical metabolic disturbances. Cardiac arrhythmias were more prevalent in the VF group, including ventricular tachycardia (32.26% vs. 2.42%, *p* < 0.001), ventricular premature beats (19.35% vs. 4.03%, *p* = 0.010), atrial fibrillation (19.35% vs. 5.65%, *p* = 0.036), and atrial premature beats (16.13% vs. 1.61%, *p* = 0.003). Medication use before admission also differed significantly, with the VF group showing higher rates of antiplatelet therapy (54.84% vs. 18.55%, *p* < 0.001) and anti-anginal therapy (35.48% vs. 18.55%, *p* = 0.042).

**Table 1A.** **Baseline characteristics for enrolled subjects**.

Characteristic		Non-VF group (n = 124)	VF group (n = 31)	*p*-value
Age (years)		63.00 (56.00, 74.50)	63.00 (55.00, 69.00)	0.318
Sex (Female, n, %)		41 (33.06%)	7 (22.58%)	0.259
MAP (mmHg)		89.00 (80.58, 99.33)	87.67 (76.83, 100.50)	0.934
Pulse (bpm)		75.46 ± 15.94	78.03 ± 17.73	0.342
Hypertension (n, %)		80 (64.52%)	26 (83.87%)	0.038
Diabetes (n, %)		46 (37.10%)	19 (61.29%)	0.015
Dyslipidemia (n, %)		81 (65.32%)	11 (35.48%)	0.002
Smoking (n, %)		56 (45.16%)	19 (61.29%)	0.108
Drinking (n, %)		43 (34.68%)	11 (35.48%)	0.933
Chronic heart failure (n, %)		2 (1.61%)	1 (3.23%)	0.491
ALT (U/L)		22.00 (14.00, 35.25)	46.00 (21.00, 64.00)	0.002
AST (U/L)		35.05 (19.18, 93.85)	126.00 (29.20, 352.30)	0.001
Creatinine (mmol/L)		78.80 (64.78, 88.28)	72.00 (46.10, 88.25)	0.336
Urea nitrogen (mmol/L)		5.19 (3.91, 6.50)	5.76 (5.05, 7.74)	0.008
Total cholesterol (mmol/L)		4.58 ± 1.03	4.56 ± 1.27	0.800
Triglycerides (mmol/L)		1.52 (1.14, 2.28)	1.66 (1.23, 2.60)	0.363
LDL-C (mmol/L)		2.65 ± 0.77	2.68 ± 0.92	0.739
HDL-C (mmol/L)		1.00 (0.90, 1.22)	0.96 (0.88, 1.20)	0.594
Medication before admission (n, %)				
	Antiplatelet	23 (18.55%)	17 (54.84%)	<0.001
	Anti-anginal	23 (18.55%)	11 (35.48%)	0.042
	Beta-blocker	13 (10.48%)	1 (3.23%)	0.304
	CCB	41 (33.06%)	7 (22.58%)	0.259
	ACEI/ARB/ARNI	21 (16.94%)	7 (22.58%)	0.465
	Diuretic	2 (1.61%)	1 (3.23%)	0.491
	Statin	20 (16.13%)	7 (22.58%)	0.397
Metabolic acidosis (n, %)		3 (2.42%)	8 (25.81%)	<0.001
Hypokalemia (n, %)		14 (11.29%)	14 (45.16%)	<0.001
Ventricular premature beats (n, %)		5 (4.03%)	6 (19.35%)	0.010
Ventricular tachycardia (n, %)		3 (2.42%)	10 (32.26%)	<0.001
Atrial fibrillation (n, %)		7 (5.65%)	6 (19.35%)	0.036
Atrial premature beats (n, %)		2 (1.61%)	5 (16.13%)	0.003
Atrial tachycardia (n, %)		0 (0.00%)	2 (6.45%)	0.039
Atrioventricular block (n, %)		4 (3.23%)	4 (12.90%)	0.085

VF, ventricular fibrillation; MAP, mean arterial pressure; ALT, alanine aminotransferase; AST, aspartate aminotransferase; LDL-C, low-density lipoprotein cholesterol; HDL-C, high-density lipoprotein cholesterol; CCB, calcium channel blocker therapy; ACEI/ARB/ARNI, angiotensin-converting enzyme inhibitor/angiotensin II receptor blocker/angiotensin receptor neprilysin inhibitor.

### 4.2 Clinical Characteristics and Coagulation Parameters

Table 1B highlights significant differences in clinical characteristics, treatment modalities, and coagulation parameters between the VF group (n = 31) and the non-VF group (n = 124). Patients in the VF group had a higher prevalence of STEMI (83.87% vs. 60.48%, *p* = 0.015) and more severe heart failure, with 48.39% classified as Killip class IV compared to only 5.65% in the non-VF group (*p* < 0.001). The VF group also showed significantly lower usage rates of key medications after admission, including aspirin (61.29% vs. 95.16%, *p* < 0.001), clopidogrel or ticagrelor (58.06% vs. 85.48%, *p* < 0.001), statins (51.61% vs. 85.48%, *p* < 0.001), beta-blockers (25.81% vs. 70.16%, *p* < 0.001), angiotensin-converting enzyme inhibitor/angiotensin II receptor blocker/angiotensin receptor neprilysin inhibitor (ACEI/ARB/ARNI) (25.81% vs. 63.71%, *p* < 0.001), and nitrates (12.90% vs. 31.45%, *p* = 0.039). Inflammatory and coagulation markers were notably elevated in the VF group, including neutrophil-to-lymphocyte ratio (NLR) (5.38 (2.88, 9.03) vs. 3.87 (2.49, 5.73), *p* = 0.046), white blood cell count (11.21 (7.11, 14.01) vs. 8.25 (6.52, 11.39), *p* = 0.025), and fibrinogen degradation products (FDP) (3.20 (2.08, 5.00) vs. 2.30 (1.90, 3.00), *p* = 0.024).

**Table 1B.** **Diagnosis, medication, and coagulation function for enrolled subjects**.

Characteristic		Non-VF group, n = 124	VF group, n = 31	*p*-value
STEMI		75 (60.48%)	26 (83.87%)	0.015
Killip grade (n, %)				<0.001
	I	87 (70.16%)	11 (35.48%)	
	II	25 (20.16%)	4 (12.90%)	
	III	5 (4.03%)	1 (3.23%)	
	IV	7 (5.65%)	15 (48.39%)	
Time of hospitalization (days)		7.56 (1.62, 62.70)	4.00 (2.00, 7.00)	0.256
Medication after admission (n, %)				
	Aspirin	118 (95.16%)	19 (61.29%)	<0.001
	Clopidogrel or ticagrelor	106 (85.48%)	18 (58.06%)	<0.001
	Statin	106 (85.48%)	16 (51.61%)	<0.001
	Beta-blockers	87 (70.16%)	8 (25.81%)	<0.001
	CCB	21 (16.94%)	1 (3.23%)	0.095
	Diuretics	18 (14.52%)	2 (6.45%)	0.369
	ACEI/ARB/ARNI	79 (63.71%)	8 (25.81%)	<0.001
	Nitrates	39 (31.45%)	4 (12.90%)	0.039
NLR		3.87 (2.49, 5.73)	5.38 (2.88, 9.03)	0.046
White blood cell (×10^12^/L)		8.25 (6.52, 11.39)	11.21 (7.11, 14.01)	0.025
Neutrophil (×10^12^/L)		6.19 (4.44, 7.91)	7.78 (4.70, 11.31)	0.114
Lymphocyte (×10^12^/L)		1.64 (1.23, 2.30)	1.57 (1.18, 2.29)	0.785
hs-CRP (mg/L)		5.90 (1.74, 19.32)	11.38 (4.20, 24.24)	0.081
INR		1.00 (0.96, 1.20)	1.10 (0.98, 1.37)	0.155
APTT (s)		25.30 (23.33, 27.70)	25.80 (23.35, 27.15)	0.800
Antithrombin III (%)		85.50 (69.08, 93.38)	83.70 (76.05, 91.15)	0.802
Prothrombin time (s)		11.60 (11.10, 12.33)	11.50 (11.10, 12.05)	0.690
Prothrombin time activity (%)		80.45 (13.90, 95.90)	86.00 (14.90, 93.95)	0.771
FDP (mg/L)		2.30 (1.90, 3.00)	3.20 (2.08, 5.00)	0.024
Fibrinogen (g/L)		2.70 (2.28, 3.36)	3.12 (2.41, 4.07)	0.262
D-dimer (mg/L)		0.60 (0.50, 0.90)	0.90 (0.50, 1.30)	0.114

VF, ventricular fibrillation; STEMI, ST-segment elevation myocardial infarction; CCB, calcium channel blocker therapy; ACEI/ARB/ARNI, angiotensin-converting enzyme inhibitor/angiotensin II receptor blocker/angiotensin receptor neprilysin inhibitor; NLR, neutrophil-to-lymphocyte ratio; hs-CRP, high-sensitivity C-reactive protein; INR, international normalized ratio; APTT, activated partial thromboplastin time; FDP, fibrinogen degradation products.

### 4.3 Interventions and Outcomes

Table 1C reveals significant differences in clinical characteristics, interventions, and outcomes between the VF group (n = 31) and the non-VF group (n = 124) during AMI. The VF group exhibited more severe coronary artery involvement, with a higher prevalence of VF-related vessels in the right coronary artery (RCA) (48.39% vs. 27.42%, *p* = 0.009) and significantly elevated Gensini scores (109.50 (84.30, 147.00) vs. 83.25 (64.50, 114.13), *p* = 0.002), indicating more extensive coronary artery disease. The use of thrombus aspiration (41.94% vs. 16.13%, *p* = 0.002) and intra-aortic balloon pumps (IABP) (25.81% vs. 2.42%, *p* < 0.001) was markedly higher in the VF group, reflecting the need for more aggressive interventions. The VF group also required significantly more frequent tracheal intubation (35.48% vs. 0.00%, *p* < 0.001), mechanical ventilation (41.94% vs. 0.00%, *p* < 0.001), and defibrillation (74.19% vs. 0.00%, *p* < 0.001), highlighting the critical nature of their condition. Cardiac biomarkers were markedly elevated in the VF group, with significantly higher levels of peak troponin I (36.45 (18.43, 41.60) vs. 4.06 (1.21, 9.60), *p* < 0.001) and CK-MB mass (112.30 (62.30, 228.70) vs. 23.60 (5.00, 85.53), *p* < 0.001), indicating severe myocardial damage. In terms of outcomes, the VF group experienced drastically higher rates of major adverse cardiac events (MACEs) (100.00% vs. 8.87%, *p* < 0.001), cardiac death (51.61% vs. 1.61%, *p* < 0.001), cardiogenic shock (54.84% vs. 4.03%, *p* < 0.001), malignant arrhythmias (96.77% vs. 4.84%, *p* < 0.001), and gastrointestinal bleeding (12.90% vs. 0.00%, *p* = 0.001).

**Table 1C.** **The in-hospital prognosis for enrolled subjects**.

Characteristic		Non-VF group, n = 124	VF group, n = 31	*p*-value
VF-related vessels (n, %)				0.009
	LM	0 (0.00%)	1 (3.23%)	
	LAD	68 (54.84%)	14 (45.16%)	
	LCX	22 (17.74%)	1 (3.23%)	
	RCA	34 (27.42%)	15 (48.39%)	
Gensini score		83.25 (64.50, 114.13)	109.50 (84.30, 147.00)	0.002
CCC (n, %)		24 (19.35%)	2 (6.45%)	0.085
Stent placement (n, %)		103 (83.06%)	24 (77.42%)	0.465
Thrombus aspiration (n, %)		20 (16.13%)	13 (41.94%)	0.002
IABP (n, %)		3 (2.42%)	8 (25.81%)	<0.001
Tracheal intubation (n, %)		0 (0.00%)	11 (35.48%)	<0.001
Mechanical ventilation (n, %)		0 (0.00%)	13 (41.94%)	<0.001
Defibrillation (n, %)		0 (0.00%)	23 (74.19%)	<0.001
Chest compression (n, %)		0 (0.00%)	16 (51.61%)	<0.001
Peak troponin I (ng/L)		4.06 (1.21, 9.60)	36.45 (18.43, 41.60)	<0.001
CK-MB mass (ng/L)		23.60 (5.00, 85.53)	112.30 (62.30, 228.70)	<0.001
Creatine kinase (U/L)		196.00 (155.00, 5853.00)	1282.00 (194.50, 3657.50)	0.479
Peak NT-proBNP (pg/mL)		1119.00 (416.25, 2972.50)	1549.00 (412.50, 2718.00)	0.899
LVEF (%)		61.00 (52.00, 66.00)	58.00 (51.00, 63.00)	0.141
E/A ratio		0.83 (0.67, 1.24)	0.81 (0.70, 1.29)	0.807
MACE (n, %)		11 (8.87%)	31 (100.00%)	<0.001
Cardiac death		2 (1.61%)	16 (51.61%)	<0.001
Cardiogenic shock		5 (4.03%)	17 (54.84%)	<0.001
Acute in-stent thrombosis		0 (0.00%)	1 (3.23%)	0.200
Recurrent myocardial infarction		1 (0.81%)	2 (6.45%)	0.102
Malignant arrhythmias		6 (4.84%)	30 (96.77%)	<0.001
Stroke		0 (0.00%)	1 (3.23%)	0.200
Gastrointestinal bleeding		0 (0.00%)	4 (12.90%)	0.001

VF, ventricular fibrillation; LM, left main coronary artery; LAD, left anterior descending artery; LCX, left circumflex artery; RCA, right coronary artery; CCC, coronary artery collateral circulation; IABP, intra-aortic balloon pumps; CK-MB, creatine kinase-MB; NT-proBNP, N-terminal pro-B-type natriuretic peptide; LVEF, left ventricular ejection fraction; E/A ratio, E peak value to A peak ratio; MACE, major adverse cardiovascular event.

### 4.4 Variable Selection and Predictive Analysis

**Fig. 2.**
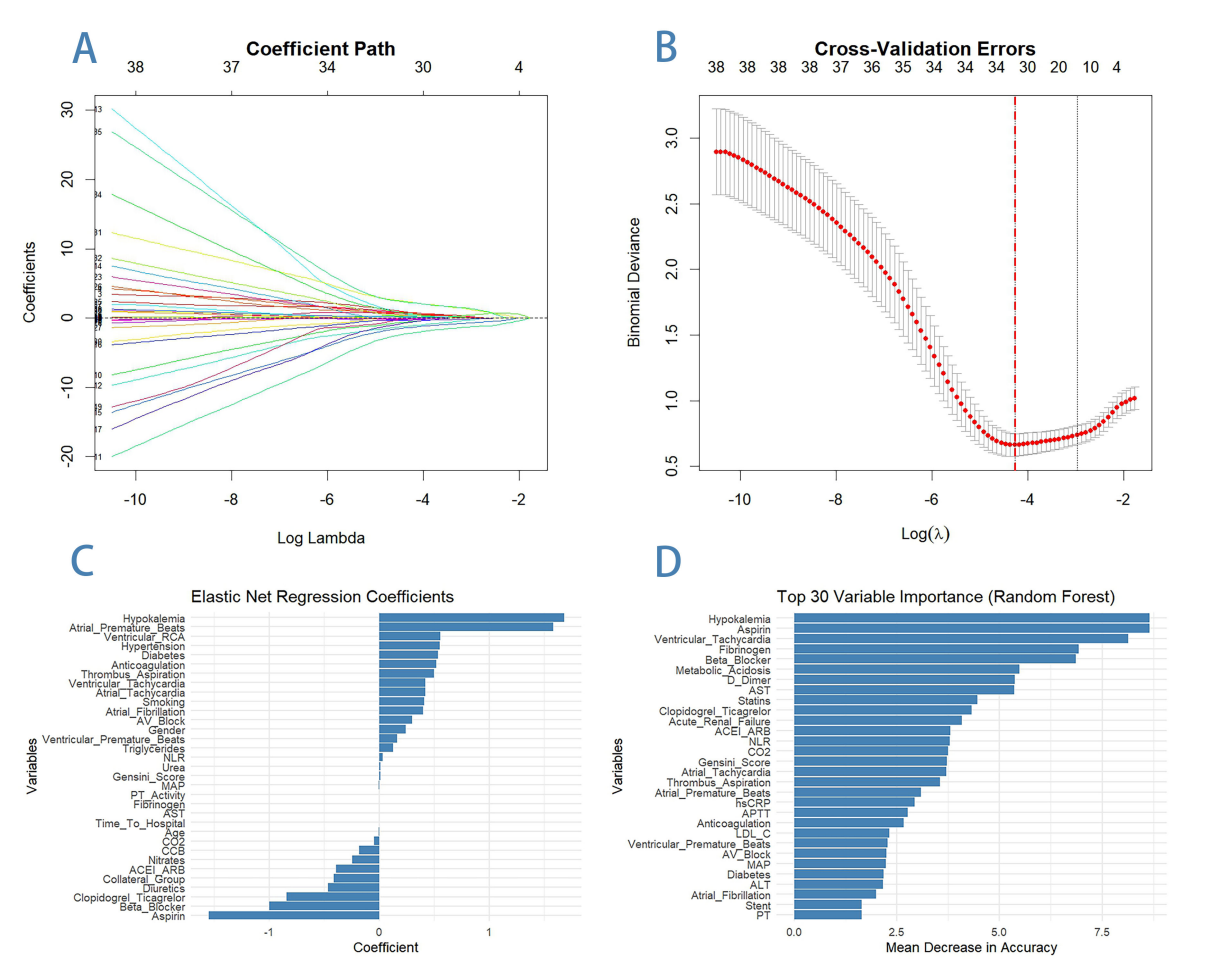
**Variable selection methods—elastic net, random forest mean decrease accuracy, and LASSO**. This figure presents the results of three variable selection methods—LASSO regression, elastic net regression, and random forest—used to identify significant predictors of ventricular fibrillation (VF). In the LASSO regression analysis (A,B), the left panel (A) illustrates the coefficient paths for variables as the regularization parameter (log lambda) changes. As lambda increases, more coefficients shrink to zero, leaving only the most important predictors, ensuring model simplicity while retaining predictive accuracy. The right panel (B) shows the cross-validation errors for different lambda values, with the optimal lambda (indicated by the dashed line) minimizing the binomial deviance. Elastic net regression results (C) are displayed as a bar plot of regression coefficients, where positive coefficients indicate variables associated with an increased risk of VF (e.g., hypokalemia and intervention on the RCA), while negative coefficients represent protective factors (e.g., beta-blockers and aspirin). Elastic net combines the strengths of LASSO and ridge regression, allowing for the retention of correlated variables while reducing overfitting. The random forest analysis (D) ranks the top 30 variables based on their importance, measured by the mean decrease in accuracy. Variables such as hypokalemia, aspirin, and ventricular tachycardia are identified as the most important predictors, highlighting their strong influence on VF risk. RCA-related ventricular fibrillation was included, it ranked 42nd in the random forest plot but was ranked higher in two other methods. Random forest is particularly valuable for capturing complex, non-linear relationships and interactions between variables, making it a powerful complement to regression-based methods. Together, these three approaches provide a robust framework for identifying and prioritizing predictors of VF. RCA, right coronary artery; NLR, neutrophil-to-lymphocyte ratio; MAP, mean arterial pressure; LDL-C, low-density lipoprotein cholesterol; PT, prothrombin time; AST, aspartate aminotransferase; CO_2_, carbon dioxide; ARB, angiotensin II receptor blockers; ACEI, angiotensin-converting enzyme inhibitors; Beta blocker, beta-adrenergic blocking agents; FDP, fibrin degradation products; LASSO, least absolute shrinkage and selection operator; CCB, calcium channel blocker therapy; AV, atrioventricular; APTT, activated partial thromboplastin time; hs-CRP, high-sensitivity C-reactive protein.

The study employed three complementary variable selection methods—elastic net, random forest, and LASSO regression—to identify significant predictors of VF from 56 variables (Fig. 2). Based on the intersection of these methods, the following nine variables were selected for further analysis: intervention on RCA, Gensini score, mean arterial pressure (MAP), aspirin, clopidogrel/ticagrelor, beta-blocker, ACEI/ARB/ARNI, NLR, and diabetes. These variables were subsequently evaluated using univariable and multivariable logistic regression analyses to determine their predictive power and quantify their association with VF risk.

The multivariable logistic regression analysis (Table 2) identified several significant predictors of VF. Intervention on the RCA was associated with a higher risk of VF (OR = 3.185, 95% CI: 1.088–9.804, *p* = 0.037). Similarly, the Gensini score (OR = 1.020, 95% CI: 1.007–1.033, *p* = 0.003) and NLR (OR = 1.149, 95% CI: 1.053–1.265, *p* = 0.002) were significant predictors, indicating that increased coronary artery disease severity and elevated systemic inflammation were associated with a greater likelihood of VF. Conversely, the use of beta-blockers (OR = 0.168, 95% CI: 0.054–0.472, *p* = 0.001) demonstrated a protective effect against VF, while the presence of diabetes (OR = 3.676, 95% CI: 1.365–10.668, *p* = 0.012) was associated with an increased risk of VF.

**Table 2.** **Logistic regression for predictors of ventricular fibrillation**.

Variable (Unit)	Intersection after variable selection			Logistic regression			
	Elastic net coefficient	Random forest mean decrease accuracy	LASSO coefficient	OR (95% CI) univariable	*p* univariable	OR (95% CI) multivariable	*p* multivariable
Intervention on RCA (Yes/No)	0.5564	8.6530	0.5622	2.824 (1.259–6.393)	0.012	3.185 (1.088–9.804)	0.037
Gensini score	0.0127	6.9297	0.0147	1.015 (1.006–1.026)	0.002	1.020 (1.007–1.033)	0.003
MAP (mmHg)	0.0036	5.4814	–1.8124	1.003 (0.980–1.026)	0.769		
Aspirin (Yes/No)	–1.5461	5.3576	0.1605	0.081 (0.025–0.232)	0.000		
Clopidogrel or ticagrelor (Yes/No)	–0.8390	4.3152	–0.8799	0.235 (0.098–0.565)	0.001		
Beta blocker (Yes/No)	–0.9985	6.8634	–1.1238	0.148 (0.057–0.348)	0.000	0.168 (0.054–0.472)	0.001
ACEI/ARB/ARNI (Yes/No)	–0.3876	3.7964	–0.2351	0.198 (0.077–0.463)	0.000		
NLR (ratio)	0.0350	3.7834	0.0358	1.124 (1.051–1.219)	0.002	1.149 (1.053–1.265)	0.002
Diabetes (Yes/No)	0.5343	2.1750	0.1174	2.685 (1.209–6.172)	0.017	3.676 (1.365–10.668)	0.012

RCA, right coronary artery; MAP, mean arterial pressure; ACEI/ARB/ARNI, angiotensin-converting enzyme inhibitor/angiotensin II receptor blocker/angiotensin receptor neprilysin inhibitor; NLR, neutrophil-to-lymphocyte ratio; LASSO, least absolute shrinkage and selection operator.

**Fig. 3.**
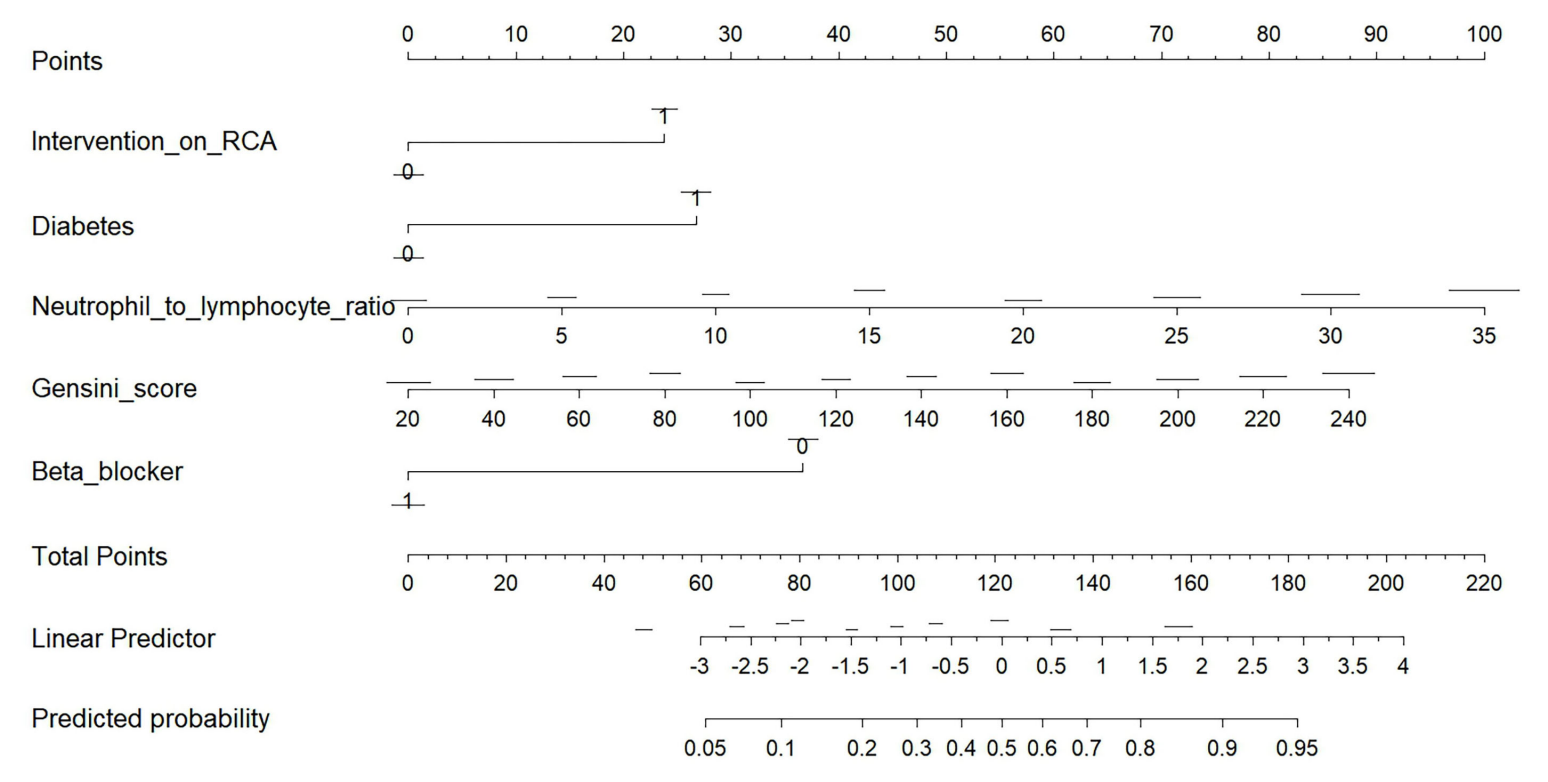
**A nomogram was constructed to facilitate the prediction of VF during PCI**. This nomogram serves as a visual tool designed to predict the probability of a specific outcome, such as the risk of a clinical event. It combines multiple predictors into a single scoring system, enabling individualized risk assessment. Each predictor variable is represented on a separate scale, with its corresponding value mapped to a “Points” scale at the top. The variables included in this nomogram are as follows: Intervention on RCA: Indicates whether an intervention was performed on the RCA. Binary values (0 or 1) contribute different points to the total score. Diabetes: Represents the presence or absence of diabetes, with binary values (0 or 1) contributing to the score. NLR: The ratio of neutrophils to lymphocytes, ranging from 0 to 35. Higher values contribute more points, reflecting increased systemic inflammation. Gensini score: A measure of coronary artery disease severity, ranging from 20 to 240. Higher scores indicate more severe disease and contribute more points. Beta blocker: Indicates whether a beta-blocker is being used, with binary values (0 or 1) contributing to the score. To use the nomogram, the value of each variable is located on its respective scale, and the corresponding points are determined by projecting upward to the “Points” scale. The points for all variables are then summed to calculate the Total Points, which are mapped to the linear predictor and subsequently to the predicted probability at the bottom of the nomogram. RCA, right coronary artery; PCI, percutaneous coronary intervention; NLR, neutrophil-to-lymphocyte ratio; VF, ventricular fibrillation.

In the study, after identifying significant predictors through regression analysis, we developed a nomogram to predict the risk of VF in patients (Fig. 3). The variables included in the nomogram are diabetes, NLR, intervention on the RCA, Gensini score, and the use of beta-blockers. These variables were selected based on their statistical significance and clinical relevance in influencing VF risk. The nomogram provides a user-friendly, visual tool that integrates these predictors into a scoring system, allowing clinicians to calculate an individualized risk score for VF.

**Fig. 4.**
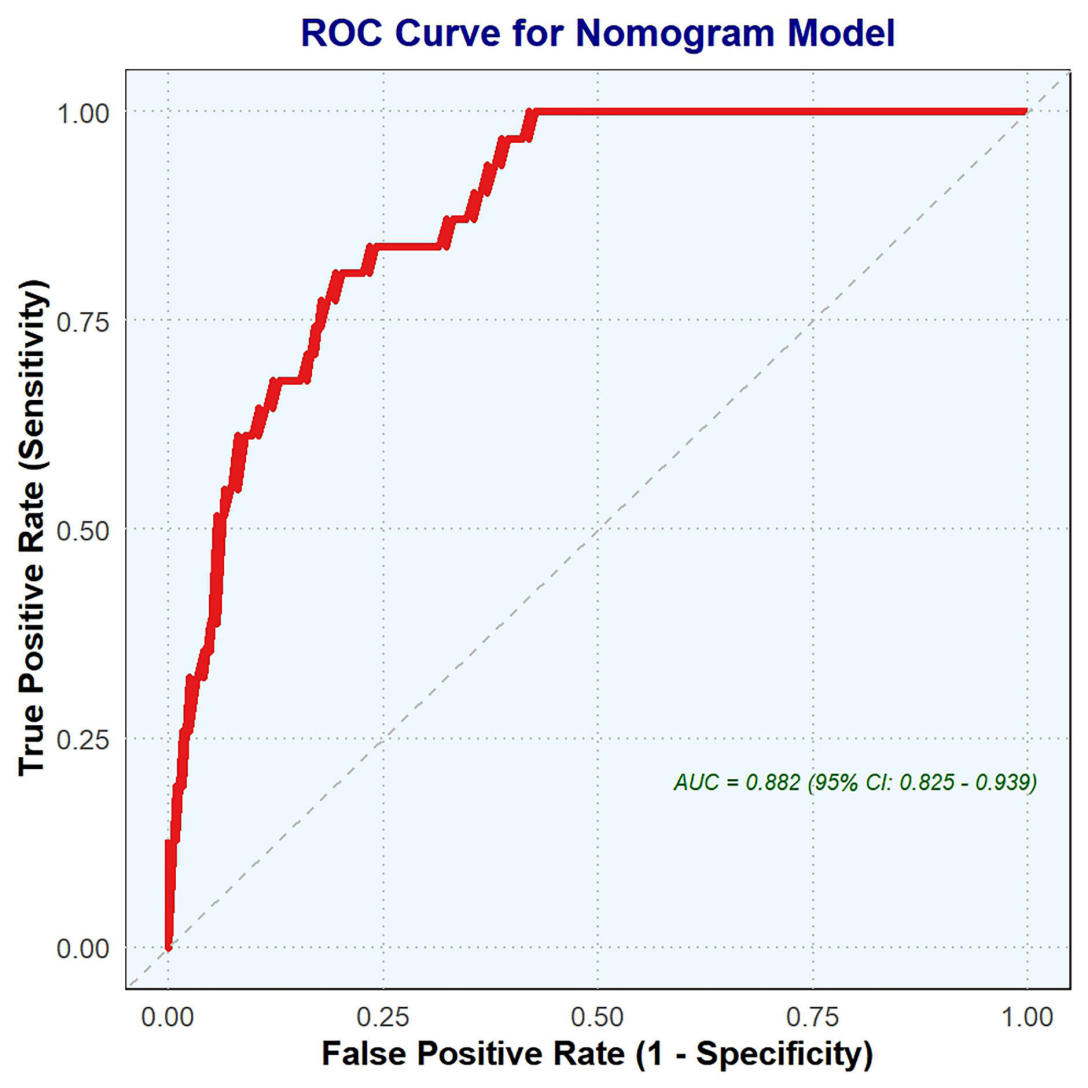
**ROC curve was employed to validate the nomogram**. This figure presents the ROC curve for the nomogram model, illustrating its diagnostic performance. The curve demonstrates a strong ability to distinguish between positive and negative outcomes, with an area under the ROC curve (AUC) of 0.882 (95% CI: 0.825–0.939). This high AUC value indicates excellent predictive accuracy, as the model achieves a good balance between sensitivity (true positive rate) and specificity (1-false positive rate). The curve’s proximity to the top-left corner further highlights the model’s robust discriminative power. ROC, receiver operating characteristic.

The ROC curve was utilized to evaluate the nomogram’s discriminative ability (Fig. 4). This analysis yielded an area under the ROC curve (AUC) of 0.882, with a 95% CI ranging from 0.825 to 0.939, indicating good accuracy. The optimal threshold, or best cutoff value, was determined to be 93.054, which balances the true positive rate and false positive rate. At this threshold, the model demonstrated a specificity of 80.65% and a sensitivity of 80.65%, reflecting a balanced performance. Additionally, the positive predictive value and negative predictive value were 51.02% and 94.34%, respectively. The Youden index, which summarizes the test’s effectiveness, was calculated to be 0.613. Collectively, these metrics underscore the model’s effectiveness in distinguishing between conditions and highlight its potential utility in clinical applications or further research.

**Fig. 5.**
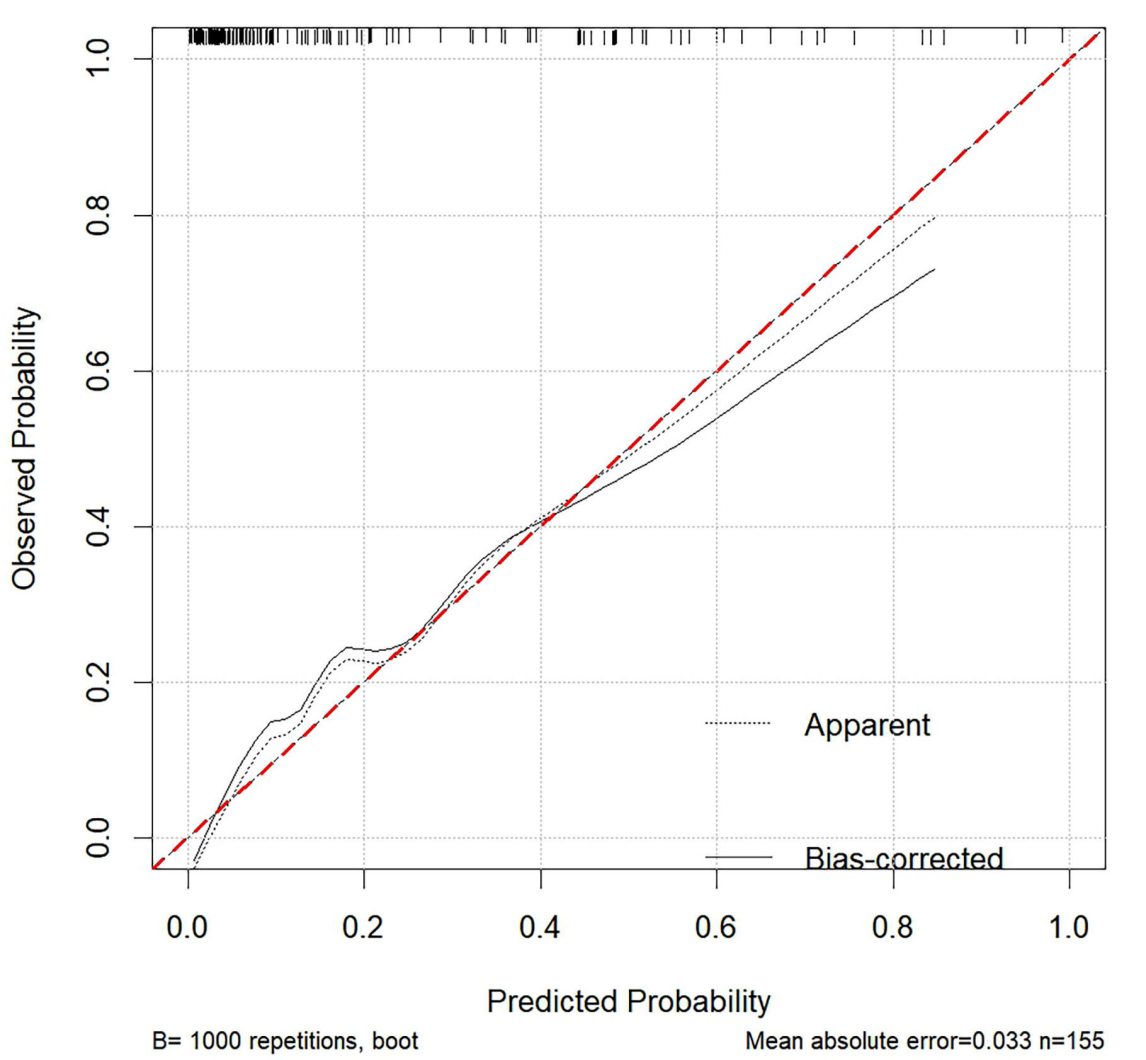
**A calibration curve was employed to validate the nomogram**. This calibration plot evaluates the agreement between the predicted probabilities and the observed outcomes for the nomogram model. The red dashed line represents the ideal calibration line, where predictions perfectly match the observed probabilities. The black solid line shows the bias-corrected performance of the model after 40 bootstrap repetitions, while the dotted line represents the apparent performance without correction. The close alignment of the bias-corrected line with the ideal line indicates good calibration, suggesting that the model’s predictions are reliable. The mean absolute error of 0.033 further supports the model’s accuracy in predicting outcomes.

The calibration curve shown in Fig. 5 was employed to assess the agreement between the predicted probabilities and observed outcomes, demonstrating the model’s accuracy in prediction: The Hosmer-Lemeshow test resulted in a statistic of 4.861 and a significant *p*-value of 0.769, confirming the model’s reliability and well fit between observed outcomes and predictions for practical use.

## 5. Discussion

The present study identified key predictors of VF during PCI in patients with AMI, a life-threatening complication that significantly increases the risk of sudden cardiac death [10, 11]. Early identification of high-risk patients and implementation of preventive measures are essential for improving outcomes. This study includes the use of a well-defined AMI cohort undergoing PCI, comprehensive data collection, and robust statistical methods, including LASSO regression, elastic net, and random forest, to identify independent predictors of VF. Our analysis revealed that diabetes, NLR, intervention on the RCA, Gensini score, and absence of beta blocker use were independent predictors of VF during PCI. These findings align with previous studies that have identified similar risk factors for ventricular arrhythmias in AMI [12, 13, 14].

Diabetes was strongly associated with VF risk, likely due to its contribution to metabolic derangements, autonomic dysfunction, and myocardial structural changes, all of which enhance arrhythmogenic potential [15, 16]. The chronic hyperglycemic state in diabetic patients can lead to increased oxidative stress and inflammation, which may disrupt ion channel function and promote electrical instability in cardiac tissues. Furthermore, diabetes is associated with alterations in cardiac autonomic regulation, which can exacerbate the risk of arrhythmias during acute ischemic events.

The NLR, a marker of systemic inflammation, has been linked to adverse outcomes in AMI and may reflect pro-arrhythmic effects mediated by inflammatory pathways [17, 18]. Elevated NLR indicates a heightened inflammatory response, which can contribute to myocardial injury and fibrosis, creating a substrate for arrhythmias. Inflammatory cytokines can also affect cardiac myocyte function and promote electrical conduction abnormalities, further linking systemic inflammation to VF risk.

The increased risk associated with RCA intervention may be attributed to the larger myocardial territory at risk and the severe ischemia-induced electrical instability in this region [19, 20]. The RCA supplies blood to critical areas of the heart, and ischemia in this territory can lead to significant myocardial damage and electrical disturbances. Additionally, ischemia can trigger the release of catecholamines, which may increase myocardial excitability and the likelihood of arrhythmias.

Additionally, the Gensini score, which quantifies the severity of coronary artery disease, correlates with VF risk, likely reflecting extensive myocardial damage, ischemia, and scarring [21, 22]. The presence of significant coronary artery disease can lead to impaired myocardial perfusion and increased vulnerability to ischemia-induced electrical disturbances. The Gensini score may serve as a surrogate marker for the extent of myocardial remodeling, which includes fibrosis and scar formation, both of which are known to create a substrate for reentrant arrhythmias.

Conversely, the absence of beta-blocker use was associated with a higher risk of VF, underscoring the protective role of beta blockers in reducing myocardial oxygen demand, stabilizing electrical activity, and mitigating arrhythmogenic triggers [23, 24]. Beta-blockers can attenuate the effects of sympathetic stimulation, which is particularly important during PCI when stress and catecholamine release are heightened. This highlights the critical need for optimizing beta-blocker therapy, particularly in high-risk patients undergoing PCI, as their absence may leave the myocardium more vulnerable to ischemia-induced arrhythmias.

To address these risks, we developed a nomogram incorporating these predictors, which demonstrated excellent discriminative ability and good calibration performance. This tool provides a practical method for clinicians to stratify AMI patients by VF risk, enabling early recognition and targeted preventive strategies such as close monitoring, electrolyte correction, and antiarrhythmic therapy [25, 26]. The nomogram’s validation through ROC curve analysis and calibration plots underscores its clinical utility and potential to improve patient outcomes.

## 6. Limitations

The study has several limitations. First, its retrospective design may introduce selection bias and limit causal inferences. Second, being conducted at a single center may reduce the generalizability of the findings. Third, unmeasured confounding factors, such as genetic predispositions, may influence VF risk. Additionally, the small sample size may limit statistical power and increase the risk of overfitting despite the use of robust statistical methods. Finally, the study did not assess the impact of using the nomogram for early preventive interventions. Future research should focus on validating the nomogram in larger multicenter cohorts and assessing its clinical utility in real-world settings. Furthermore, studies exploring the impact of early preventive strategies guided by the nomogram on outcomes such as VF incidence, PCI success, and short- and long-term mortality are warranted [27]. Mechanistic studies investigating the links between identified risk factors and VF development during PCI could also uncover novel therapeutic targets and improve our understanding of VF pathophysiology.

## 7. Conclusions

This study identified diabetes, NLR, intervention on the RCA, Gensini score, and absence of beta-blocker use as key predictors of VF during PCI in AMI patients. A nomogram incorporating these factors demonstrated excellent predictive performance and good calibration. This tool can help clinicians identify high-risk patients and implement targeted preventive strategies.

## Availability of Data and Materials

The data supporting the findings of this study are available from the corresponding author upon reasonable request.
